# Women’s Quality of Life at 6 Weeks Postpartum: Influence of the Discomfort Present in the Puerperium

**DOI:** 10.3390/ijerph16020253

**Published:** 2019-01-17

**Authors:** Juan Miguel Martínez-Galiano, Antonio Hernández-Martínez, Julián Rodríguez-Almagro, Miguel Delgado-Rodríguez, Ana Rubio-Alvarez, Juan Gómez-Salgado

**Affiliations:** 1 Department of Nursing, University of Jaen, 23071 Jaen, Spain; jgaliano@ujaen.es; 2 Consortium for Biomedical Research in Epidemiology and Public Health (CIBERESP), 28029 Madrid, Spain; mdelgado@ujaen.es; 3 Mancha-Centro Hospital, Alcázar de San Juan, 13600 Ciudad Real, Spain; antonio.hmartinez@uclm.es; 4 Department of Nursing, University of Castilla la Mancha, 13071 Ciudad Real, Spain; 5 Department of Health Sciences, University of Jaen, 23071 Jaen, Spain; 6 Obstetrics Service, University Hospital of Torrejon de Ardoz, 28850 Madrid, Spain; a.rubio.alvarez@hotmail.es; 7 Department of Nursing, University of Huelva, 21007 Huelva, Spain; jgsalgad@gmail.com; 8 Safety and Health Posgrade Program, Universidad Espíritu Santo, Guayaquil 091650, Ecuador

**Keywords:** health-related quality of life, pregnancy, puerperium, maternal discomfort, postpartum problems

## Abstract

*Background*: Discomfort during the puerperium period is very frequent in the lives of women but the influence of this discomfort on the women’s quality of life has been little studied. The objective of this study is to establish the association between discomfort and frequent problems of women in the puerperium and their quality of life score. *Methods*: A cross-sectional study on postpartum Spanish women was performed. Women older than 18 years and who had had a live birth were included. Less than 1% of women refused to participate in the study. Data were collected on socio-demographic, obstetric and newborn variables, on maternal problems/ discomfort in the postnatal period and on parameters that are quality of life indicators. An ad hoc online questionnaire which included the SF-36 Health Survey was used. Crude mean difference (cMD) and adjusted mean difference (aMD) were calculated through multiple linear regression. *Results*: 2990 women participated in the study. The greater problems causing quality of life loss were depressive symptoms (aMD = −12.40, CI 95%: −10.79, −14.01), lactation problems (aMD = −4.30, CI 95%: −2.97, −5.63), problems for sexual intercourse after childbirth (aMD = −6.34, CI 95%: −5.07, −7.60) and urinary incontinence (aMD = −4.97, CI 95%: −6.30, −3.65), among others. These have been detected as risk factors that affect the quality of life of the postpartum woman. *Conclusions*: The discomfort and problems manifested in the 6 weeks after childbirth have an influence that deeply affects the quality of life of postpartum women.

## 1. Introduction

The postpartum period is a critical stage in which a series of changes in the woman occur, which impact at a physical, psychic and social level [1]. The Health-Related Quality Of Life (HRQOL), as defined by the World Health Organization (WHO), refers to an individual’s perception of their position in life taking into account the cultural context and value systems in which they live and in relation to their goals, expectations, standards, and concerns [2]. This is more and more considered as an indicator of maternal and child health, and health professionals that intervene view it as an essential priority in their care planning [3,4,5,6].

The HRQOL in women during pregnancy and in different phases of the postpartum period has been studied [4,7,8,9,10,11]. The results of a systematic review with meta-analysis that included 18 studies concluded that the type of delivery was associated with the HRQOL of the woman in the postpartum period: women with vaginal delivery had better quality of life than women who gave birth by caesarean section [7]. A study carried out in Rotterdam (The Netherlands) on 3936 mothers evaluated the HRQOL throughout pregnancy and its associated factors. Thus, anxiety was detected as a predictive factor, among others [9]. The authors of a Greek study on 154 women detected an association between postpartum depression symptoms and mothers’ quality of life [11].

Most of the studies on HRQOL and pregnancy, childbirth and the puerperium are oriented to identifying predisposing factors during pregnancy and childbirth in the quality of life of women. However, there are very scarce and inconsistent results that determine the effects the discomfort and problems women suffer in the postnatal period have on their HRQOL. After a thorough review of the literature of the last five years, few studies have been found that relate discomfort in the puerperium and HRQOL of women. In the light of this situation, and in order to be able to design appropriate health plans to meet the demands and needs of women with these inconveniences, it was proposed to establish the association between this discomfort and the most common problems women suffer in the puerperium and the HRQOL score in the first 6 weeks after childbirth.

## 2. Materials and Methods

A cross-sectional study was carried out on women who gave birth in Spain during the year 2017. This study was approved by the Ethical Committee on Clinical Research (CECI, for its Spanish acronym and ethical code 69-C) of the La Mancha-Centro Centre. Before starting the questionnaire, the participating women had to read a fact sheet about the study, its objectives, etc., and mark a box in which they showed their consent to participate in it, i.e., they signed a online informed consent (ticking the option if they wanted to participate or not). Births with antepartum stillbirths and women under 18 years of age were excluded. For the sample size estimation [12], an alpha risk of 5% and a beta risk of 10% (power = 90%) were considered, as well as the potential number of 10 predictors and an effect size of 0.02 points, estimating a minimum of 1036 women.

### 2.1. Sources of Information

A questionnaire applied to women six weeks after delivery was used for data collection. The questionnaire was elaborated by the researchers themselves with the questions that included the study variables. It contained 35 items (three open questions, 32 closed questions) on sociodemographic and clinical characteristics, obstetric outcomes, discomfort and maternal puerperal problems, and newborn data. In addition to this questionnaire, the SF-36 validated instrument [13,14] was also used to measure the quality of life. SF-36 is a generic scale (composed of 36 questions (items) that provide a profile of the state of health and is applicable to both patients and the general population). It has been useful to assess health-related quality of life (HRQOL) in the general population and in specific subgroups [13]. The questionnaire had previously been piloted and distributed to women through the main women associations and the Associations of Spanish Midwives Federation (FAME), as well as their member associations. These involved their midwives in the dissemination of the project and in the recruitment of the participants. Once the study subjects were selected and had agreed to participate, they were given the instructions to complete the questionnaire, which they filled according to their availability. There was a telephone number available to answer all the possible questions these women may have had in completing the questionnaire, as well as a discussion session to the same end. The following variables were collected:

The main dependent variables were the scores globally obtained in the SF-36 questionnaire on HRQOL and in the psychological and physical dimensions of this questionnaire.

The main independent variables were women symptoms of: constipation problems, presence of hemorrhoids, wound infection (need for professional treatment after hospital discharge and/or consumption of antibiotics, perineal pain, headache, breast pain, back pain, discomfort or burning sensation during urination, fecal incontinence (inability to control bowel movements), urinary incontinence (involuntary urine loss), tiredness, sadness, anxiety (nervousness and/or restlessness), depression (depressive mood), problems related to maintaining sexual intercourse, problems with the couple’s relationship dynamics after birth and problems related to lactation.

The independent variables that were taken into account for confounding control were of demographic and clinical type. In this way, the following factors were considered: maternal age, academic level and nationality of the mother, attendance to maternal training, gestational age at which the delivery took place, presence of health problems during pregnancy, multiple pregnancy, parity, type of delivery, episiotomy and/or severe perineal tearing (III/IV degree), type of feeding of the infant, newborn admittance, and quality of life affected by other problems and/or factors not related to the pregnancy, delivery and puerperium.

### 2.2. Statistical Analysis Employed

First, a descriptive analysis was performed using absolute and relative frequencies for categorical and mean variables with standard deviation (SD) for quantitative variables. For the determination of the relationship between the different factors and the HRQOL, the crude mean difference (cMD) of scores was calculated by means of linear regression and, later, the adjusted mean difference (aMD) was calculated by means of multiple linear regression. A *p* < 0.05 was considered as significant. All analyses were carried out with the SPSS v 24.0 statistical package (SPSS Inc., Chicago, IL, EEUU).

## 3. Results

2990 women were included in the study. 51.5% (n = 1541) of the women who gave birth were 35 years old or more, and 59.1% (n = 1767) of the participants had university studies. 96.0% (n = 2870) of the women had a singleton pregnancy and 92.2% (n = 2757) gave birth at 37 weeks of gestation or later. 36.4% (n = 1089) had an episiotomy performed, 4.2% (n = 125) had a severe tearing of III or IV degree, and 75.5% (n = 2257) of these women had a vaginal birth. 24.2% (n = 724) of these women suffered an impact on their quality of life by factors external to the process of pregnancy, childbirth and the puerperium, as can be seen in Table 1, where the characteristics of the study population are listed.

Table 2 shows that all the studied problems presented a negative and statistically significant relationship with the HRQOL in a global way due to confounding factors adjustment. Haemorrhoids had the lowest mean decline in its scores (aMD = −1.27, CI 95%: −0.02, −2.51), while the problem that increased HRQOL loss arose with depressive symptoms (aMD = −12.40, CI 95%: −10.79, −14.01).

As for the physical dimension, haemorrhoids were the only problem that did not present an association with quality of life, while the greatest decrease in HRQOL occurred in women with depressive symptomatology (aMD = −9.35, CI 95%: −7.47, −11.22). For the psychological dimension, the greatest decrease in the quality of life took place in women with depressive symptoms (aMD = −15.45, CI 95%: −13.59, −17.31). It should be noted that the discomfort affecting psychologically such as depressive symptomatology (aMD = −12.40, CI 95%: −10.79, −14.01), anxiety (aMD = −11.76, CI 95%: −10.48, −13.04) and feelings of sadness (aMD = −8.50, CI 95%: −7.29, −9.72), together with others that also have an important psychological component such as problems with the couple’s relationship dynamics (aMD = −9.75, CI 95%: −8.45, −11.05) and tiredness (aMD = −8.78, CI 95%: −7.12, −10.45), are those that implied a greater loss of total HRQOL (Table 2). In this same Table 2, the different problems and discomforts studied and their associations with the HRQOL in its physical, psychological and global dimension can also be noted.

## 4. Discussion

All the inconveniences and discomfort of women during the puerperium that have been included in our study such as haemorrhoids, constipation, wound infection at childbirth, pain (perineal, head, chest, and back), urinary discomfort, incontinence (faecal and urinary), symptoms of tiredness, sadness, anxiety and depression have been analysed as risk factors for the HRQOL of women during the puerperium. A positive relationship was also identified between women’s difficulties in maintaining sexual intercourse, problems with the couple’s relationship dynamics and lactation problems and their maternal quality of life six weeks after delivery. The discomfort and problems related to mental health such as depressive symptoms, anxiety, sadness, etc., as well as those that have an important psychological component such as problems with the couple’s relationship dynamics, are the ones that most negatively affect and that have a greater impact on the HRQOL of women during the puerperium.

### Limitations and Strengths

In the case of a selection bias associated with non-response, this has not influenced the results. Women’s response to the participation was preponderant. Only 29 refused to participate and nothing suggests that those who did not respond would have done so differently from those that did. The existence of an information bias is unlikely: the data collected, as well as the way in which the possible responses were posed, do not require a high level of education. The questions were presented in a basic and simple way, with an accessible and comprehensible language style for any educational level. It is not completely possible to reject a recall bias although the information was collected in a short interval of time. This is why, in case of an influence on the results, we believe it would have been minimal. Besides, women know perfectly their health provider and they identify whether it is public or private. They also especially remember the information received on their birth process, which is generally highly valued and deserves special attention on their part.

The general questionnaire that was used was elaborated with the variables that had been found in the literature review. In addition, a pilot study was carried out in which the questionnaire was used and which served to improve it, but it must be borne in mind that it was not validated. It was a questionnaire prepared specifically by researchers for this study as a structured instrument for collecting information. The questionnaire used to collect the information on the quality of life (Spanish version of the Health Questionnaire SF-36) was validated [13,14].

Among the strengths of the study, the following must be highlighted: the sample size is large, with women from different geographical areas and who have been assisted both in public and private hospitals. The instrument used was the SF-36 validated questionnaire [13,14,15], which has already been used in Spanish pregnant women [15]. 

Woman’s depressive symptoms have been associated with a worse HRQOL in the physical and mental dimension, as well as in their overall quality of life. This goes in line with the results found by Papamarkou et al. in the weeks or months following delivery [11], with outcomes from a French study on 150 women during postpartum [16] and with those found in the postnatal period by de Tychey et al. [17]. Subclinical depressive symptoms have been associated with alterations in the mother-child relationship during the postpartum period [18].

In our study, anxiety symptoms have been detected as a risk factor for a worse quality of life of the woman during the first six weeks of the postpartum period. A Canadian study conducted on postpartum women by Misri and Swift [19] detected an association between the generalised anxiety disorder and a worse quality of life, although it is necessary to establish differences between these results [19], where there was a diagnosis of generalised anxiety disorder with DSM-5 criteria, and our results, where women report anxiety-related symptoms and behaviours.

Lin et al. [20], in a study on 866 women, reported that urinary incontinence during postpartum had a negative impact on women’s HRQOL. These outcomes are in line with what has been identified in our study and in a systematic review that included 66 studies [21].

Having problems with sexual intercourse during the puerperium was detected as a risk factor for having a worse HRQOL. In a similar way, Lagaert et al. [22] concluded that the deterioration of the sexual function of women and the dyspareunia in the first 6 postpartum weeks impair postpartum HRQOL.

A Spanish study conducted by Triviño-Juárez et al. [23] reported that at the sixth week, breastfed children ate more, slept more, and attended the emergency medical centre in fewer occasions. In this sense, they established an indirect relationship between lactation and the quality of life of the mother, results that were also detected in our study.

According to our results, constipation, haemorrhoids, wound infection related with childbirth, different forms of pain (perineal pain, headache, back pain and breast pain), discomfort and burning during urination, faecal incontinence, feelings of tiredness, sadness and anxiety, and problems with the couple’s relationship dynamics have also been associated with a worse HRQOL In the literature we have not found any report on these discomforts and the quality of life in the puerperium. Only one study carried out in Canada in 1574 women by Mannion et al. [24] identified back pain during the puerperium with a worse quality of life, in line with our results. This is why it represents a great innovation and establishes the basis for future studies that can support or contradict these results and continue to advance the knowledge to improve women’s health.

## 5. Conclusions

The discomfort that appears in postpartum women as a result of the process of pregnancy, childbirth and the puerperium adversely affect their HRQOL. The presence of depression, anxiety, problems in the relationships of couple or headache are the discomforts that have a greater negative impact on the quality of women at 6 weeks postpartum. In the light of the results of this study, it is possible to consider measures in the clinical practice of professionals attending childbirth (avoiding practices that are associated with the incidence of specific discomfort or a greater number of these) and health professionals who come into contact with women during the postpartum period to carry out a thorough assessment of maternal discomfort and to attempt to implement both preventive and therapeutic measures to reduce maternal discomfort and thus improve quality of life in one of the most complex phases of a woman’s life.

## Figures and Tables

**Table 1:** Characteristics of the study population.

Variable	n (%)
Maternal age	
<35 years	1449 (48.5)
≥35 years	1541 (51.5)
Academic level	
No studies	7 (0.2)
Primary	146 (4.9)
Secondary	1070 (35.8)
University	1767 (59.1)
Nationality	
Spanish	2886 (96.5)
Other	104 (3.5)
Parity	
Primiparous	1503 (50.3)
Multiparous	1487 (49.7)
Maternal training attendance	
No	1200 (40.1)
Yes	1790 (59.9)
Health problems during pregnancy	
No	2113 (70.7)
Yes	877 (29.3)
Multiple pregnancy	
No	2870 (96.0)
Yes	120 (4.0)
Type of delivery	
Vaginal	2257 (75.5)
Caesarean	733 (24.5)
Episiotomy	
No	1901 (63.6)
Yes	1089 (36.4)
Acute perineal tearing (III/IV degree)	
No	2865 (95.8)
Yes	125 (4.2)
Gestational age at delivery	
≥37 weeks of gestation	2757 (92.2)
˂37 weeks of gestation	233 (7.8)
Newborn admittance	
No	2741 (70.6)
Yes	249 (8.3)
Type of feeding	
Maternal lactation	2112 (70.6)
Artiftial lactation	878 (29.4)
Quality of life affected by other problems/factors not related to the pregnancy, delivery and the puerperium	
No	2266 (75.8)
Yes	724 (24.2)

**Table 2:** Problems/discomfort a week after delivery.

Variable		SF-36 Scores		
	n (%)	Physical HRQOL Mean (SD)	Psychological HRQOL Mean (SD)	Global HRQOL Mean (SD)
**Constipation**				
No (ref.)	1743 (58.3)	77.8 (19.66)	67.9 (20.37)	72.8 (17.71)
Yes	1247 (41.7)	73.8 (21.21)	64.8 (21.27)	69.3 (18.74)
MD CI 95%		**−4.02 (−2.52, −5.51)**	**−3.09 (−1.58, −4.60)**	**−3.52 (−2.23, −4.88)**
aMD CI 95%		**−3.86 (−2.46, −5.26)**	**−2.92 (−1.48, −4.35)**	**−3.39 (−2.16, −4.62)**
**Haemorrhoids**				
No	1603 (53.6)	76.2 (20.34)	66.9 (20.68)	71.5 (18.11)
Yes	1387 (46.4)	76.1 (20.50)	66.2 (20.94)	71.1 (18.36)
MD CI 95%		−0.11 (−1.58, 1.36)	−0.74 (−2.23, 0.76)	−0.42 (−1.74, 0.89)
aMD CI 95%		−0.90 (−2.31, 0.52)	**−1.64 (−0.19, −3.08)**	**−1.27 (−0.02, −2.51)**
**Infected wound**				
No	2779 (92.9)	76.7 (20.03)	67.0 (20.58)	71.9 (17.88)
Yes	211 (7.1)	68.1 (23.53)	61.1 (22.92)	64.6 (21.16)
MD CI 95%		**−8.65 (−5.37, −11.92)**	**−5.92 (−2.72, −9.12)**	**−7.82 (−4.34, −10.23)**
aMD CI 95%		**−5.95 (−3.19, −8.71)**	**−3.02 (−0.19, −5.85)**	**−4.48 (−2.01, −6.91)**
**Perineal pain**				
No	1729 (57.8)	78.1 (19.57)	68.8 (20.37)	73.4 (17.59)
Yes	1261 (42.2)	73.5 (21.24)	63.6 (21.03)	68.5 (18.70)
MD CI 95%		**−4.61 (−2.52, −6.11)**	**−5.23 (−3.73, −6.74)**	**−4.92 (−3.61, −6.23)**
aMD CI 95%		**−5.88 (−4.30, −7.47)**	**−6.44 (−4.82, −8.06)**	**−6.16 (−4.78, −7.55)**
**Headache**				
No	2313 (77.4)	78.7 (18.99)	69.2 (19.60)	74.0 (16.87)
Yes	677 (22.6)	67.4 (22.62)	57.48 (22.21)	62.5 (19.82)
MD CI 95%		**−11.24 (−9.37, −13.11)**	**−11.77 (−9.91, −13.62)**	**−11.50 (−9.86, −13.14)**
aMD CI 95%		**−8.98 (−7.33, −10.64)**	**−9.68 (−7.99, −11.36)**	**−9.33 (−7.89, −10.77)**
**Breast pain**				
No	1728 (57.8)	78.2 (19.27)	68.9 (20.01)	73.5 (17.18)
Yes	1262 (42.2)	73.3 (21.59)	63.5 (21.46)	68.4 (19.18)
MD CI 95%		**−4.86 (−3.36, −6.35)**	**−5.38 (−3.87, −6.90)**	**−5.12 (−3.79, −6.45)**
aMD CI 95%		**−4.14 (−2.74, −5.54)**	**−4.49 (−3.06, −5.92)**	**−4.31 (−3.09, −5.54)**
**Back pain**				
No	1676 (56.1)	80.7 (17.65)	70.4 (19.34)	75.6 (16.09)
Yes	1314 (43.9)	70.3 (22.13)	61.7 (21.57)	66.0 (19.33)
MD CI 95%		**−10.50 (−9.02, −11.96)**	**−8.72 (−7.28, −10.26)**	**−9.63 (−8.33, −10.93)**
aMD CI 95%		**−9.01 (−7.64, −10.38)**	**−7.29 (−5.88, −8.71)**	**−8.15 (−6.94, −9.35)**
**Burning during urination**				
No	2430 (81.3)	77.3 (19.69)	67.7 (20.54)	72.5 (17.73)
Yes	560 (18.7)	70.9 (22.61)	61.6 (21.19)	66.3 (19.44)
MD CI 95%		**−6.37 (−4.33, −8.39)**	**−6.17 (−4.23, −8.10)**	**−6.27 (−4.51, −8.02)**
aMD CI 95%		**−6.09 (−4.31, −7.56)**	**−5.85 (−4.03, −7.66)**	**−5.97 (−4.12, −7.52)**
**Urinary incontinence**				
No	2007 (67.1)	78.0 (19.34)	68.2 (20.13)	73.2 (17.24)
Yes	983 (32.9)	72.2 (21.96)	63.1 (21.71)	67.7 (19.58)
MD CI 95%		**−5.79 (−4.18, −7.40)**	**−5.23 (−3.62, −6.85)**	**−5.51 (−4.07, −6.95)**
aMD CI 95%		**−5.24 (−3.73, −6.75)**	**−4.71 (−3.16, −6.25)**	**−4.97 (−3.65, −6.30)**
**Faecal incontinence**				
No	2844 (95.1)	76.5 (20.24)	66.8 (20.76)	71.6 (18.11)
Yes	146 (4.9)	69.5 (22.64)	62.0 (21.29)	65.7 (19.57)
MD CI 95%		**−7.00 (−3.22, −10.77)**	**−4.82 (−1.26, −8.38)**	**−5.91 (−2.64, −9.18)**
aMD CI 95%		**−5.15 (−1.87, −8.42)**	−1.45 (0.35, −6.36)	**−4.08 (−1.20, −6.96)**
**Fatigue**				
No	459 (15.4)	83.2 (16.70)	76.6 (17.89)	79.9 (15.10)
Yes	2531 (84.6)	74.8 (20.76)	64.8 (20.78)	69.8 (18.31)
MD CI 95%		**−8.39 (−6.66, −10.12)**	**−11.85 (−10.02, −13.68)**	**−10.12 (−8.56, −11.68)**
aMD CI 95%		**−7.08 (−5.17, −8.99)**	**−10.49 (−8.56, −12.42)**	**−8.78 (−7.12, −10.45)**
**Sadness**				
No	1648 (55.1)	79.4 (18.90)	72.1 (18.07)	75.8 (16.06)
Yes	1342 (44.9)	72.1 (21.46)	59.8 (21.89)	65.9 (19.23)
MD CI 95%		**−7.34 (−5.90, −8.79)**	**−12.38 (−10.93, −13.82)**	**−9.86 (−8.59, −11.15)**
aMD CI 95%		**−5.94 (−4.53, −7.35)**	**−11. 07 (−9.67, −12.47)**	**−8.50 (−7.29, −9.72)**
**Anxiety**				
No	2049 (68.5)	79.6 (18.31)	72.0 (17.77)	75.8 (15.63)
Yes	941 (31.5)	68.48 (22.57)	54.8 (22.04)	61.6 (19.63)
MD CI 95%		**−11.16 (−9.51, −12.81)**	**−17.21 (−15.60, −16.82)**	**−14.19 (−12.75, −15.61)**
aMD CI 95%		**−8.60 (−7.09, −10.10)**	**−14.92 (−13.44, −16.39)**	**−11.76 (−10.48, −13.04)**
**Depression**				
No	2485 (83.1)	78.2 (19.11)	69.6 (18.99)	73.9 (16.66)
Yes	505 (16.9)	65.9 (23.37)	51.6 (22.77)	58.8 (20.24)
MD CI 95%		**−12.25 (−10.07, −14.42)**	**−18.04 (−15.92, −20.16)**	**−15.14 (−13.49, −16.80)**
aMD CI 95%		**−9.35 (−7.47, −11.22)**	**−15.45 (−13.59, −17.31)**	**−12.40 (−10.79, −14.01)**
**Sexual problems**				
No	1815 (60.7)	74.3 (16.87)	69.9 (19.34)	74.3 (16.87)
Yes	1175 (39.5)	72.2 (21.46)	61.4 (21.91)	66.8 (19.29)
MD CI 95%		**−6.50 (−4.98, −8.02)**	**−8.51 (−6.97, −10.05)**	**−7.51 (−6.16, −8.85)**
aMD CI 95%		**−5.42 (−3.97, −6.87)**	**−7.25 (−5.78, −8.72)**	**−6.34 (−5.07, −7.60)**
**Couple’s relationship**				
No	2092 (70.0)	78.8 (19.18)	71.0 (18.33)	74.9 (16.42)
Yes	898 (30.0)	69.9 (21.83)	56.2 (22.48)	63.08 (19.50)
MD CI 95%		**−8.87 (−7.22, −10.52)**	**−14.81 (−13.14, −16.35)**	**−11.84 (−10.38, −13.30)**
aMD CI 95%		**−6.78 (−5.27, −8.30)**	**−12.72 (−11.23, −14.22)**	**−9.75 (−8.45, −11.05)**
**Lactation problems**				
No	1897 (63.4)	77.9 (19.34)	69.0 (19.96)	73.5 (17.25)
Yes	1093 (36.6)	73.0 (21.81	62.4 (21.59)	67.7 (19.29)
MD CI 95%		**−4.96 (−3.40, −6.52)**	**−6.53 (−4.97, −8.10)**	**−5.74 (−4.36, −7.13)**
aMD CI 95%		**−3.45 (−1.93, −4.98)**	**−5.15 (−3.60, −6.69)**	**−4.30 (−2.97, −5.63)**

Adjusted by maternal age, academic level, nationality, maternal training, gestational age, problems during pregnancy, multiple delivery, parity, type of delivery, episiotomy, acute tearing, type of lactation, newborn admittance and quality of life affected by other problems/factors not related to the pregnancy, delivery and the puerperium. Bold data mean statistically significant differences.

## References

[1] Ko Y. (2004). Postpartum fatigue. Hu Li Za Zhi.

[2] Group W. (1995). The World Health Organization Quality of Life assessment (WHOQOL): Position paper from the World Health Organization. Soc. Sci. Med..

[3] Mogos M.F., August E.M., Salinas-Miranda A.A., Sultan D.H., Salihu H.M. (2013). A Systematic Review of Quality of Life Measures in Pregnant and Postpartum Mothers. Appl. Res. Qual. Life.

[4] Calou C.G.P., de Oliveira M.F., Carvalho F.H.C., Soares P.R.A.L., Bezerra R.A., de Lima S.K.M., Antezana F.J., de Souza Aquino P., Castro R.C.M.B., Pinheiro A.K.B. (2018). Maternal predictors related to quality of life in pregnant women in the Northeast of Brazil. Health Qual. Life Outcomes.

[5] Oviedo-Caro M.A., Bueno-Antequera J., Munguía-Izquierdo D. (2018). Explanatory factors and levels of health-related quality of life among healthy pregnant women at midpregnancy: A cross-sectional study of The PregnActive Project. J. Adv. Nurs..

[6] Ozdemir M.E., Cilingir I.U., Ilhan G., Yildiz E., Ohanoglu K. (2018). The effect of the systematic birth preparation program on fear of vaginal delivery and quality of life. Arch. Gynecol. Obstet..

[7] Rezaei N., Tavalaee Z., Sayehmiri K., Sharifi N., Daliri S. (2018). The relationship between quality of life and methods of delivery: A systematic review and meta-analysis. Electron Physician.

[8] Van den Bosch A.A.S., Goossens M., Bonouvrié K., Winkens B., Nijhuis J.G., Roumen F.J.M.E., Wassen M.M.L.H. (2018). Maternal quality of life in routine labor epidural analgesia versus labor analgesia on request: Results of a randomized trial. Qual. Life Res..

[9] Bai G., Raat H., Jaddoe V.W.V., Mautner E., Korfage I.J. (2018). Trajectories and predictors of women’s health-related quality of life during pregnancy: A large longitudinal cohort study. Montazeri A, editor. PLoS ONE.

[10] Park S., Choi N.-K. (2018). The relationships between timing of first childbirth, parity, and health-related quality of life. Qual. Life Res..

[11] Papamarkou M., Sarafis P., Kaite C.P., Malliarou M., Tsounis A., Niakas D. (2017). Investigation of the association between quality of life and depressive symptoms during postpartum period: A correlational study. BMC Womens Health.

[12] Cohen J. (1977). Statistical Power Analysis for the Behavioral Sciences.

[13] Vilagut G., Ferrer M., Rajmil L., Rebollo P., Permanyer-Miralda G., Quintana J.M., Santed R., Valderas J.M., Ribera A., Domingo-Salvany A. (2018). The Spanish version of the Short Form 36 Health Survey: A decade of experience and new developments. Gac Sanit.

[14] Alonso J., Regidor E., Barrio G., Prieto L., Rodríguez C., de la Fuente L. (1998). Population reference values of the Spanish version of the Health Questionnaire SF-36. Med. Clin..

[15] Triviño-Juárez J.-M., Romero-Ayuso D., Nieto-Pereda B., Forjaz M.-J., Criado-Álvarez J.-J., Arruti-Sevilla B., Avilés-Gamez B., Oliver-Barrecheguren C., Mellizo-Díaz S., Soto-Lucía C. (2017). Health related quality of life of women at the sixth week and sixth month postpartum by mode of birth. Women Birth.

[16] Cherif R., Feki I., Gassara H., Baati I., Sellami R., Feki H., Chaabene K., Masmoudi J. (2017). Post-partum depressive symptoms: Prevalence, risk factors and relationship with quality of life. Gynécol. Obs. Fertil. Sénol..

[17] De Tychey C., Briançon S., Lighezzolo J., Spitz E., Kabuth B., de Luigi V., Messembourg C., Girvan F., Rosati A., Thockler A. (2008). Quality of life, postnatal depression and baby gender. J. Clin. Nurs..

[18] Braarud H.C., Skotheim S., Høie K., Markhus M.W., Kjellevold M., Graff I.E., Berle J.Ø., Stormark K.M. (2017). Affective facial expression in sub-clinically depressed and non-depressed mothers during contingent and non-contingent face-to-face interactions with their infants. Infant Behav. Dev..

[19] Misri S., Swift E. (2015). Generalized Anxiety Disorder and Major Depressive Disorder in Pregnant and Postpartum Women: Maternal Quality of Life and Treatment Outcomes. J. Obstet. Gynaecol. Can..

[20] Lin Y.-H., Chang S.-D., Hsieh W.-C., Chang Y.-L., Chueh H.-Y., Chao A.-S., Liang C.C. (2018). Persistent stress urinary incontinence during pregnancy and one year after delivery; its prevalence, risk factors and impact on quality of life in Taiwanese women: An observational cohort study. Taiwan J. Obstet. Gynecol..

[21] Van der Woude D.A.A., Pijnenborg J.M.A., de Vries J. (2015). Health status and quality of life in postpartum women: A systematic review of associated factors. Eur. J. Obstet. Gynecol. Reprod. Biol..

[22] Lagaert L., Weyers S., Van Kerrebroeck H., Elaut E. (2017). Postpartum dyspareunia and sexual functioning: A prospective cohort study. Eur. J. Contracept. Reprod. Health Care.

[23] Triviño-Juárez J.M., Nieto-Pereda B., Romero-Ayuso D., Arruti-Sevilla B., Avilés-Gámez B., Forjaz M.J., Oliver-Barrecheguren C., Mellizo-Díaz S., Soto-Lucía C., Plá-Mestre R. (2016). Quality of life of mothers at the sixth week and sixth month postpartum and type of infant feeding. Midwifery.

[24] Mannion C.A., Vinturache A.E., McDonald S.W., Tough S.C. (2015). The Influence of Back Pain and Urinary Incontinence on Daily Tasks of Mothers at 12 Months Postpartum. Schooling CM, editor. PLoS ONE.

